# PREPARING FUTURE HEALTH PROFESSIONALS FOR AGE, CULTURAL, AND TECHNOLOGY (ACT) FRIENDLY CARE IN HEALTH SCIENCES

**DOI:** 10.1093/geroni/igae098.3881

**Published:** 2024-12-31

**Authors:** Nosaiba Rayan-Gharra, Shoshi Keisari, Lia Ring, Shelly Engdau, Dikla Segel-Karpas, Anna Zisberg, Alexandra Danial-Saad

**Affiliations:** University of Haifa, Haifa, Hefa, Israel; University of Haifa, Haifa, Hefa, Israel; Ashkelon Academic College, Ashkilon, HaDarom, Israel; University of Haifa, Haifa, Hefa, Israel; University of Haifa, Haifa, Hefa, Israel; University of Haifa, Haifa, Hefa, Israel; University of Haifa, Haifa, Hefa, Israel

## Abstract

The aging population emphasizes the need for tailored training in welfare and health sciences, particularly for students who will work with culturally diverse older populations using advanced technological tools. This study examines how well the curriculum at the Faculty of Welfare and Health Sciences prepares students for Age, Cultural, and Technology (ACT) Friendly care. An online survey was conducted among 208 faculty lecturers, teaching mandatory undergraduate and/or graduate courses (BA only=65, MA only=82, both BA and MA=61). Respondents rated how extensively they address aging, multiculturalism, and technology in their courses. Only 15.4% had received training in aging studies, while 16.4% were involved in aging research. Aging-related topics were included in 31% of undergraduate and 27% of graduate courses. Multiculturalism was similarly integrated (28% for both programs), while technology was less addressed (13% for BA and 8% for MA courses). Only 17.3% of respondents extensively incorporated ACT topics in their teaching, and 65.7% did not address them at all. Multivariate analysis controlling for gender, seniority, and research area showed that involvement in aging, multiculturalism, or technology research predicted teaching these topics (p< 0.05). The findings highlight a significant gap in preparing students for ACT-friendly care, particularly at the graduate level, and a lack of integration of the three domains. This study underscores the need for ongoing assessment and curriculum development to equip future health professionals for age-appropriate, culturally sensitive, and technologically integrated care. Faculty development initiatives are also recommended to enhance educators’ capacity to incorporate these critical areas into their teaching.

